# Clinical effects of HBV infection on patients with Rheumatoid Arthritis and Systemic Lupus Erythematosus

**DOI:** 10.12669/pjms.39.5.7232

**Published:** 2023

**Authors:** Lin Duan, Chao Yang, Tingting Cai, Wei Li

**Affiliations:** 1 Lin Duan, Department of Nuclear Medicine, Affiliated Hospital of Hebei University, Baoding 071000, Hebei, China; 2 Chao Yang, Department of Nuclear Medicine, Affiliated Hospital of Hebei University, Baoding 071000, Hebei, China; 3 Tingting Cai, Department of Nuclear Medicine, Affiliated Hospital of Hebei University, Baoding 071000, Hebei, China; 4 Wei Li, Department of Nuclear Medicine, Affiliated Hospital of Hebei University, Baoding 071000, Hebei, China

**Keywords:** HBV infection, Rheumatoid arthritis, Systemic lupus erythematosus, Disease activity, Immune function

## Abstract

**Objective:**

To investigate the clinical effects of HBV infection on patients with rheumatoid arthritis (RA) and systemic lupus erythematosus (SLE).

**Methods:**

This is a prospective study. Thirty patients with RA and 30 patients with SLE admitted to Affiliated Hospital of Hebei University from January 2020 to December 2021 with co-infection of HBV were randomly selected and divided into two groups. Both groups were given anti-HBV treatment. An additional 60 patients with a healthy physical examination during the same period were also selected as a control group. The disease activity, immune function and serum inflammatory factor levels were compared between the RA group and the SLE group before and after treatment.

**Results:**

After anti-HBV treatment, DAS scores in the RA group and SLEDAI scores in the SLE group were significantly lower than before treatment(*P*<0.05). The levels of IgG, IgA, IgM and CD8+ in the RA group and the SLE group after treatment were significantly lower than those before treatment. The levels of CCP, RF, ESR and CRP in the RA group before and after treatment were higher than those in the control group(*P*<0.05). The levels of ESR and CRP in the SLE group before and after treatment were higher than those in the control group, with statistically significant differences(*P*<0.05).

**Conclusion:**

Patients in the RA and SLE groups after HBV infection have an increased degree of inflammatory response in their organism, an altered normal state of immunoglobulin and T-lymphocyte subsets, and a loss of organism immune function, leading to an increase in disease activity.

## INTRODUCTION

Hepatitis B Virus (HBV), as a common clinical hepatotropic DNA virus, may cause chronic hepatitis B once infected.^1^,^2^ Most of the patients are in a decompensated stage when they are treated, and some severe cases have already suffered from liver cancer.^3^ The occurrence and progression of HBV have a close bearing on the immune tolerance of the body. Rheumatoid arthritis (RA) and systemic lupus erythematosus (SLE), as autoimmune diseases, are closely related to the immune system of the body.

According to a clinical study^4^, patients usually suffer from RA and SLE after viral infection, which is attributed to the destruction of the immune system and the aggravation of immune dysfunction in such patients after co-infection with HBV.^5^ Additionally, various immunosuppressive agents, such as glucocorticoids and biologics, are clinically applied in the treatment of RA and SLE, which may increase the risk of HBV infection or contribute to the reactivation of HBV in the latent phase.^6^ For the clarification of the effect of HBV infection on patients with RA and SLE, anti-HBV was used in this study to treat patients with RA and SLE co-infected with HBV in order to provide guidance for clinical treatment. The effects of HBV infection on disease activity, immune function and serum inflammatory factors in RA and SLE were observed to provide a theoretical basis for guiding the clinical selection of a reasonable treatment regimen.

## METHODS

This is a prospective study, which included 120 participants in total in this study, thirty patients with RA and 30 patients with SLE admitted to Affiliated Hospital of Hebei University from January 2020 to December 2021 with co-infection of HBV were randomly selected and divided into two groups: the RA group and SLE group. An additional 60 patients with a healthy physical examination during the same period were also selected as a control group.

### Ethical Approval:

The study was approved by the Institutional Ethics Committee of Affiliated Hospital of Hebei University (No.:2022020; date: December 30, 2022), and written informed consent was obtained from all participants.

### Inclusion criteria:

- Patients with RA meeting the diagnostic criteria of the “Guidelines for Diagnosis and Treatment of Rheumatoid Arthritis” and SLE meeting the diagnostic criteria of the “Guidelines for Diagnosis and Treatment of Systemic Lupus Erythematosus”;
- Patients with complete clinical data.

### Exclusion criteria:

- Patients with malignant tumors;
- Patients with incomplete clinical data;
- Patients with other autoimmune diseases;
- Patients with decompensated acute liver injury caused by other reasons.

Both the RA group and SLE group were given conventional antiviral, symptomatic and supportive treatment for six months. And case data collection ceased in June 2021.

### Observation indicators:

Five markers of hepatitis B were detected and HBV-DNA was quantitatively detected. Disease activity assessment: The 28-joint Disease Activity Score (DAS) was used to assess the disease activity of patients with RA. The SLE Disease Activity Index (SLEDAI) was applied to assess the disease activity of patients with SLE. *Immunological indicators:* The levels of immunoglobulins IgG, IgA, and IgM in serum, and the levels of T-cell subsets CD4+ and CD8+ and the ratio of CD4+/CD8+ were detected. *Serum indicators:* Anti-cyclic citrullinated peptide (CCP), rheumatoid factor (RF), erythrocyte sedimentation rate (ESR), and C-reactive protein (CRP) were measured. In the SLE group, only ESR and CRP were measured.

### Statistical methods:

All data in this study were statistically analyzed using SPSS22.0 software. Measurement data were expressed as mean ± standard deviation (*χ̅*±*S*), and t test was used for comparison among the groups. Enumeration data were expressed as n (%), and c² test was used for comparison among the groups. *P*<0.05 indicates a statistically significant difference.

## RESULTS

No statistically significant difference was observed between the RA and SLE groups and the control group in terms of gender, age, weight and BMI (*P*>0.05), which was comparable. See Table-I. The levels of five items of hepatitis B and HBV-DNA in the RA group and the SLE group were significantly different from those in the control group (*P*<0.05). Table-II.

**Table-I:** Comparison of general information [n (%), (*χ̅*±*S*)].

Item	RA group (n=30)	SLE group (n=30)	Control group (n=60)
Gender (Male/Female)	20/10	22/8	42/18^*,Δ^
Age (yrs)	49.27±5.43	48.57±4.35	47.15±4.83^*,Δ^
Body weight (kg)	67.64±7.55	69.57±6.19	68.62±5.49^*,Δ^
BMI (kg/m^2^)	24.17±4.02	23.59±4.04	24.56±3.97^*,Δ^

***Note:*** * Indicates *P*>0.05 compared with the RA group; ^Δ^ indicates *P*>0.05 compared with the SLE group.

**Table-II:** Comparison of the levels of five items of hepatitis B and HBV-DNA among the three groups [(*χ̅*±*S*)].

Item	RA group (n=30)	SLE group (n=30)	Control group (n=60)
HbsAg (ng/ml)	72.20±3.30	71.66±3.74*	0.31±0.08^Δ^
HBsAb (mIU/ml)	3.80±1.07	3.44±1.11*	144.71±34.84^Δ^
HBeAg (PEIU /ml)	5.94±1.79	5.91±1.13*	0.40±0.09^Δ^
HBeAb (PEIU /ml)	10.08±1.19	10.01±1.24*	0.15±0.07^Δ^
HBcAb (PEIU /ml)	148.11±23.65	150.76±25.97*	0.37±0.09^Δ^
HBV-DNA (×10^3^IU /ml)	82.34±16.87	83.12±16.30*	0.61±0.30^Δ^

*Note:*

* Indicates P>0.05 compared with the RA group;

Δ indicates *P*>0.05 compared with the RA group and the SLE group.

The DAS score of the RA group after treatment was significantly lower than that before treatment, with a statistically significant difference (*P*<0.05). The SLEDAI score of the SLE group after treatment was significantly lower than that before treatment, with a statistically significant difference (*P*<0.05). Table-III.

**Table-III:** Comparison of disease activity (*χ̅*±*S*).

Item	Before treatment	After treatment	T value	P value
DAS28 score (point)	2.66±0.39	2.36±0.40	2.900	0.005
SLEDAI score (point)	13.50±2.08	7.30±2.02	11.712	0.000

The levels of IgG, IgA, IgM and CD8+ in the RA group and the SLE group after treatment were significantly lower than those before treatment, and the levels of CD3+, CD4+ and CD4+/CD8+ were significantly higher than those before treatment, with statistically significant differences (*P*<0.05). Table-IV. Table-VI.

**Table-IV:** Comparison of immune indexes between the RA group and the control group before and after treatment (*χ̅*±*S*).

Item	RA group		Control group

	Before treatment	After treatment	
IgG (g/L)	12.20±0.10*	4.53±2.27^*,Δ^	3.60±1.93
IgA (g/L)	3.56±0.16*	2.80±0.70^*,Δ^	1.46±0.27
IgM (g/L)	3.34±0.21*	2.80±0.73^*,Δ^	1.34±0.73
CD3^+^(%)	33.01±5.95*	52.57±7.02^*,Δ^	65.21±5.81
CD4^+^(%)	27.38±4.66*	37.73±5.32^*,Δ^	42.83±4.75
CD8^+^(%)	41.46±4.19*	32.09±4.22^*,Δ^	27.19±3.45
CD4^+^/CD8^+^	0.66±0.06*	1.18±0.09^*,Δ^	1.58±0.16

***Note:***

* Indicates P<0.05 compared with the control group;

Δ indicates P<0.05 compared with before treatment.

**Table-V:** Comparison of serum inflammatory factors between the RA group and the control group before and after treatment (*χ̅*±*S*).

Item	RA group		Control group

	Before treatment	After treatment	
CCP (U/mL)	16.35±5.23*	6.43±2.22^*,Δ^	5.50±2.52
RF (U/mL)	22.49±4.31*	10.12±3.07^*,Δ^	7.80±3.57
ESR (mm/h)	61.35±7.89	20.08±6.64	13.28±2.24
CRP (mg/mL)	61.35±7.89*	20.08±6.64^*,Δ^	13.28±4.24

***Note:***

* Indicates P<0.05 compared with a control group;

Δ indicates P<0.05 compared with before treatment.

**Table-VI:** Comparison of immune indicators between the SLE group and the control group before and after treatment [n (%), (*χ̅*±*S*)].

Item	SLE group		Control group

	Before treatment	After treatment	
IgG(g/L)	12.12±0.18*	7.60±2.55^*,Δ^	3.60±1.93
IgA(g/L)	3.19±0.30*	2.80±0.70^*,Δ^	1.46±0.27
IgM(g/L)	3.08±0.24*	2.50±0.44^*,Δ^	1.34±0.73
CD3^+^(%)	39.06±4.52*	48.88±5.10^*,Δ^	65.21±5.81
CD4^+^(%)	29.70±3.41*	39.39±4.27^*,Δ^	42.83±4.75
CD8^+^(%)	38.21±4.19*	31.29±4.69^*,Δ^	27.19±3.45
CD4^+^/CD8^+^	0.78±0.05*	1.27±0.08^*,Δ^	1.58±0.16

*Note:*

* Indicates P<0.05 compared with the control group;

Δ indicates *P*<0.05 compared with before treatment.

The levels of CCP, RF, ESR and CRP in the RA group before and after treatment were higher than those in the control group, with statistically significant differences (*P*<0.05). The intra-group comparison showed that the levels of CCP, RF, ESR and CRP in the RA group after treatment were significantly lower than those before treatment, with statistically significant differences (*P*<0.05). Table-V.

The levels of ESR and CRP in the SLE group before and after treatment were higher than those in the control group, with statistically significant differences (*P*<0.05). The intra-group comparison showed that the levels of ESR and CRP in the SLE group after treatment were significantly lower than those before treatment, with a statistically significant difference (*P*<0.05). Table-VII.

**Table-VII:** Comparison of inflammatory factors between the SLE group and the control group before and after treatment [n (%), (*χ̅*±*S*)].

Item	SLE group		Control group

	Before treatment	After treatment	
ESR (mm/h)	66.29±5.35*	27.36±5.16^*,Δ^	13.28±4.24
CRP (mg/mL)	24.04±4.78*	16.94±3.83^*,Δ^	12.02±3.02

***Note:***

* Indicates P<0.05 compared with the control group;

Δ indicates P<0.05 compared with before treatment.

## DISCUSSION

It was found in this study that the DAS28 scores of RA patients with HBV infection decreased after treatment, and that of SLE patients also decreased after treatment, indicating that HBV infection can aggravate the disease activity of RA and SLE patients; Not only that, the disease activity of RA and SLE patients was reduced after anti-HBV treatment, indicating that anti-HBV treatment of RA and SLE patients with HBV infection has obvious clinical efficacy in controlling RA and SLE. These are consistent with the results of several clinical studies.^7^ To explain the reason, HBV aggravates the inflammatory response in patients with RA and SLE, supplemented by biologics for the treatment of RA and SLE, which can lead to aggravation or even worsening of hepatitis B disease.

The interaction of these factors resulted in increased disease activity in RA and SLE patients during HBV decompensation. In response to this, entecavir clears HBV and contributes to a decrease in disease activity.^8^ IgM, IgA and IgG are important players in humoral immunity.^9^ CD3+ responds to the overall cellular immune status of the body, and CD4+ T lymphocytes direct the body against HBV. In contrast, CD8+ T lymphocytes are able to recognize HBV-infected target cells and kill them, ultimately clearing HBV.^10^ Meanwhile, CD8+ stimulates B cells and T cells to secrete inflammatory factors and destroy bones. CD4+/CD8+ responds to the immune status of the body, and when HBV infection occurs, it suffers from a lower ratio, decreased immune function of the patient, and impaired anti-HBV ability of the body.^11^

HBV infection is a serious public health problem currently spreading in China.^12^,^13^ Most patients with HBV infection are only clinically diagnosed when they have obvious clinical manifestations, such as spider nevus and hepatosplenomegaly, and some of them are already in the decompensated stage of HBV infection.^14^ RA and SLE, which are autoimmune diseases, both require the application of immunosuppressive drugs in their treatment, combined with glucocorticoids if necessary. To date, there are few clinical studies on the effects of HBV infection on disease activity, immune function and inflammatory response in RA and SLE, let alone any clinical reports on the effects before and after treatment.

It was shown in this study that for RA and SLE patients with co-infection with HBV, the levels of IgG, IgA, IgM and CD8+ were significantly lower after treatment, while CD3+, CD4+ and CD4+/CD8+ were significantly higher, indicating that in RA and SLE patients with co-infection with HBV, their immunoglobulin and T lymphocyte subsets recovered with the improvement of their disease. For this reason, clinical monitoring of the levels of immunoglobulin and T lymphocyte subsets is a good indicator of the patient’s disease activity.

CCP is a highly specific autoantibody secreted by B cells, which can be used to predict the severity of RA disease. It is positive in the early stage of RA and shows good sensitivity and specificity for the diagnosis of RA. It can also be used to predict the severity of RA disease.^15^,^16^ RF is an autoantibody that uses IgG as a target antigen, and its secretion increases in the presence of RA, which binds to IgG to produce a reaction.^17^ In this study, both CCP and RF levels were higher in RA patients than in the normal population before and after treatment.

It has been shown in related studies^18^ that the occurrence of cirrhosis in HBV infection has a close bearing on the release of inflammatory factors such as serum ESR and CRP. CRP is an acute temporal protein^19^ involved in the non-specific immune response of the body. It has been pointed out that the level of CRP concentration is positively correlated with the severity of inflammation in the body.^20^

In this study, RA and SLE patients with co-infection with HBV had significantly higher ESR and CRP levels than the normal population. After treatment, these patients showed disease improvement, lower levels of the organismal inflammatory response, and correspondingly lower ESR and CRP levels, but still higher than the normal population. The conclusion of this study provides a new clinical reference for the clinical impact of HBV infection on patients with SLE and RA.

### Limitations:

It includes small sample size, short follow-up time, and failure to combine the study content with the final patient regression.

## CONCLUSION

HBV infection significantly aggravates the disease in patients with RA and SLE. RA and SLE patients with HBV co-infection suffer from an increased disease activity, a loss of body immune status, an altered normal state of immunoglobulin and T-lymphocyte subsets, and a loss of organism immune function, and an increased degree of inflammatory response.

### Authors’ Contributions:

**LD and CY:** Carried out the studies, participated in collecting data, drafted the manuscript, are responsible and accountable for the accuracy and integrity of the work.

**TC:** Performed the statistical analysis and participated in its design.

**WL:** Participated in acquisition, analysis, or interpretation of data and draft the manuscript. All authors read and approved the final manuscript.
